# From recognition to expression: extending cardiovascular emotional dampening to facial expressions under elevated blood pressure

**DOI:** 10.3389/fpsyt.2025.1681377

**Published:** 2025-11-05

**Authors:** Shatabdi Bhowmick, Meenakshi Shukla, Rakesh Pandey

**Affiliations:** ^1^ Department of Psychology, University of Allahabad, Allahabad, India; ^2^ Department of Psychology, Banaras Hindu University, Varanasi, India

**Keywords:** hypertension, cardiovascular emotional dampening, facial expressions, facial action coding system (FACS), blood pressure

## Abstract

**Introduction:**

Cardiovascular Emotional Dampening (CED) refers to blunted emotional responsiveness in individuals with elevated blood pressure (BP), but research has exclusively focused on how such individuals perceive others’ emotions. Given evidence that the ability to produce facial expressions of emotions is closely tied to emotion recognition via shared neural mechanisms, examining expressive deficits in CED could reveal additional pathways linking elevated BP with emotional communication. This study examined whether individuals with higher systolic and diastolic BP exhibit reduced accuracy and intensity when generating prototypical facial expressions of emotion.

**Methods:**

Participants (N = 74) across normotensive (n=33), prehypertensive (n=21), and hypertensive (n=20) categories were instructed to pose six basic emotions. Facial Action Units (AUs) were coded using certified human coders and OpenFace, allowing comparison of AU intensities, human-machine agreement, and expression accuracy. Prototypical emotion templates were used to determine accuracy, and interrater agreement was quantified via five complementary indices.

**Results:**

Emotional expression accuracy was significantly lower in prehypertensive and hypertensive groups, particularly for sadness, fear, and surprise. Correlational analyses revealed significant negative associations of SBP and DBP with accuracy of expressing sadness, disgust, and anger. Notably, expressions of happiness were preserved. Although overall agreement between human and machine ratings was high, reduced intensity and increased AU sparsity at higher BP levels likely suppressed reliability metrics.

**Discussion:**

These findings extend the CED framework from recognition to expression, revealing that elevated BP may blunt the expressive channel for particularly negative emotions, irrespective of arousal level. Though facial expression of emotions is limited to negative emotions, it is generalized across different levels of arousal within this category - from low (sad), moderate (disgust), to high (anger).The pattern suggests central autonomic influences on facial expressivity of emotions and opens new directions for identifying emotional communication deficits in at risk populations.

## Introduction

Over the past two decades, the term “Cardiovascular Emotional Dampening” (CED) has been used to describe reduced emotional responsiveness in individuals with higher resting blood pressure (BP) (1–6). In CED, negative stimuli are perceived as less negative, and positive stimuli are experienced as less positive (3). This dampening effect has been linked to elevated BP levels, even when still within the normal range (1–3). For instance, Pury and her associates discovered a negative relationship between participants’ self-reported emotional responses to visually presented emotions and BP (3). Likewise, another team of researchers (1) reported similar results when they explored the generalizability of this phenomenon in an older high-risk population of African Americans. Elevated BP and higher peripheral resistance were linked to weaker recognition of emotional content in both faces and written narratives. Another study conducted on an all-male sample by McCubbin et al. (2) found a negative correlation between the accuracy of emotion recognition and resting diastolic blood pressure (DBP). Furthermore, even within the normotensive range, individuals with a parental history of hypertension have been found to exhibit reduced emotional responsiveness to both positive and negative stimuli compared to those with normotensive parents (7).

Since these initial studies, researchers have further explored CED, producing several new insights into its nature and expression. Interestingly, in contrast to these emerging research findings on the linear inverse relationship between BP and emotion recognition, thereby supporting the CED phenomenon, one particularly intriguing study explored and found support for a possible curvilinear relationship between BP and CED (8). Moreover, existing evidence on CED has primarily focused on consciously recognized emotions in a single, usually visual, modality. However, research indicates that CED extends beyond visual stimuli to auditory and cross-modal emotional cues (4, 5).

Several studies have demonstrated that individuals with elevated blood pressure, including prehypertensives and hypertensives, show reduced accuracy in recognizing emotions from faces, voices, and even body gestures (4, 5, 9). Evidence also suggests that CED is not limited to explicit emotion recognition but affects implicit processing, as shown through behavioral and psychophysiological measures (4, 5, 10). While CED generally reflects blunted emotional responsiveness, some recent findings indicate heightened reactivity to threat-related cues, such as angry faces, among individuals with hypertension (11). This implies that hypertension may heighten sensitivity to threat-related cues rather than dampen all emotions, revealing a complex, context-dependent link between BP and emotion. Shukla & Pandey (6) investigated how participants’ startle response was impacted by the relative predominance of their present subjective positive mood over negative mood. A recent work has further expanded the scope of CED to the olfactory domain, showing that elevated blood pressure affects the perception of pleasant and unpleasant odors (12).

While most studies on CED have focused on impairments in emotion recognition, the ability to express emotions accurately is an equally essential component of socio-emotional functioning. Recognition and expression are reciprocal: understanding others is only one side of communication; expressing one’s own feelings through facial and bodily cues enables others to respond appropriately (13, 14). Expression also serves a self-regulatory function, influencing how emotions are internally experienced through feedback mechanisms linking facial musculature and autonomic activity (15, 16). The communicative role of facial expressions has been extensively theorized in contemporary social-affective models. Within the Theory of Affective Pragmatics (TAP; 17), emotional expressions are viewed as communicative acts that do more than mirror internal states. They also serve directive and declarative functions, shaping how others interpret situations and respond behaviorally. Complementing this, the Emotions as Social Information (EASI) model (18, 19) emphasizes the social consequences of expressed emotions, proposing that facial and bodily cues disambiguate social contexts and guide observers through affective reactions and inferential processes. Together, these frameworks position emotional expression as a vital channel through which emotions regulate interpersonal understanding and coordination, rather than as a mere by-product of feeling. Difficulty producing clear expressions may weaken interpersonal responsiveness, cause emotional miscommunication, and reduce social connectedness. Extending the CED framework from emotion recognition to emotional expression can thus provide a more complete understanding of how elevated BP may disrupt the bidirectional flow of emotional information within social contexts.

Beyond social-communicative accounts, classic embodiment and facial-feedback theories provide a mechanistic rationale for why emotional expression may change under elevated blood pressure. The facial-feedback hypothesis, dating back to Darwin (20) and James (21), proposes that activity in facial muscles contributes causally to emotional experience (e.g., smiling can make one feel happier, frowning sadder) (meta-analysis, 22, 23). Embodiment theory extends this principle, suggesting that both recognizing and producing emotions rely on the partial re-enactment of bodily states associated with prior affective experiences (16, 24). Since elevated BP has been shown to dampen autonomic and sensorimotor responsiveness, for instance, through reduced baroreflex-related cortical modulation and lower autonomic flexibility (25, 26), these alterations may weaken feedback loops linking bodily and emotional states, leading to less vivid or less accurate facial expressions. This framework grounds our hypothesis that expressive dampening may represent another manifestation of CED.

The emotion expression aspect is critical to investigate, as it could offer valuable insights into the neural mechanisms underlying CED. A large body of research highlights the role of mirror neurons, which are activated both when performing and when observing an action, in emotional perception. For instance, perceiving a facial expression of disgust in someone else and experiencing that emotion oneself engage overlapping neural circuits (27). Evidence from clinical populations supports this connection: children with Moebius syndrome (a congenital condition characterized by the inability to produce facial emotional expressions), show marked deficits in recognizing emotions in others (28). Similarly, patients with schizophrenia or Parkinson’s disease, both of whom are impaired in generating facial expressions, also demonstrate difficulties in emotion recognition (29–31). Experimental studies on healthy individuals further confirm this link, as blocking facial mimicry (e.g., by holding a pen in the mouth) disrupts the recognition of emotions from both faces and bodies. Conversely, interventions designed to train facial expression mimicry have been shown to enhance emotion recognition in clinical groups (32, 33). Together, this evidence suggests that an individual’s ability to produce facial emotional expressions plays a key role in perceiving emotions in others. Thus, examining whether individuals with elevated BP exhibit deficits in producing facial expressions of emotions is important. Such deficits could implicate dysfunctions in the mirror neuron system, further explaining the mechanisms underlying CED.

Building on the gaps identified in previous literature, the present study aims to investigate whether CED is also evident in the production of facial emotional expressions among individuals with elevated BP. Prior work has consistently documented diminished emotion recognition in individuals with elevated BP (e.g. 1, 4, 5, 11). While no study, to our knowledge, has yet measured expressive deficits in the same populations, it is plausible that expressive blunting may accompany recognition deficits as another manifestation of reduced affective reactivity under physiological dysregulation. This study represents the first attempt at exploring how expression of emotions in faces is altered among individuals with elevated BP. Specifically, we examine group differences (normotensives, prehypertensives, hypertensives) and continuous links between BP and the accuracy and intensity of facial expressions. We hypothesized that higher BP would be associated with reduced accuracy and intensity of posed facial expressions, particularly for negative emotions.

## Method

### Ethics approval

The study protocol and procedures were approved by the Institutional Ethics Review Board of University of Allahabad (Ref. No.: IERB/34/2025, Study IERB ID: 2025-04DOPSY).

### Participants

In this study, the recruited participants (both male and female) were in the age range of 18–65 years and were classified into either one of the three BP categories of normotensives (individuals with normal BP), prehypertensives (individuals whose blood pressure is higher than that of normotensives, yet not high enough to be categorized as hypertensives), and hypertensives (individuals with clinically elevated BP) based on their BP averaged over a total of six readings taken on two consecutive days. *A priori* power analysis calculation (using G*Power 3.1) revealed that for a moderate effect size of .50, an alpha of 0.05, a power of 0.95, and number of covariates equal to 3 (age, gender, education level), the required sample size for conducting a partial correlation would be 38. Similarly, for groupwise comparisons of accuracy and intensity of facial emotion expressions using one-way analysis of covariance (ANCOVA), *a priori* sample size calculation with an effect size of .50, an alpha of 0.05, a power of 0.95, number of groups equal to 3, numerator df equal to 2, and number of covariates equal to 3 (age, gender, education level), revealed a sample size of 66. Thus, more than 70 participants were targeted for recruitment to allow for missing data and/or outliers. Notably, the effect size of .50 used in calculating the required sample size was informed by prior CED studies, where the effect size varied widely between.016 (6) to.86 (5).

As per the BP ranges outlined in the Seventh Report of the Joint National Committee on Prevention, Detection, Evaluation, and Treatment of High Blood Pressure (JNC-7; 34), normotensives have their systolic blood pressure (SBP) below 120 mmHg and their diastolic blood pressure (DBP) below 80 mmHg); prehypertensives have their SBP between 120–139 mmHg and/or DBP between 80–89 mmHg; while hypertensives (stage 1) are those with their SBP between 140–159 mmHg and/or DBP is 90–99 mmHg.

Individuals who were capable of understanding and reading at least basic Hindi were included. A medical co-morbidity screening questionnaire was used to determine if participants had any physical and/or mental health issues. Participants were not included in this study if they had a thyroid condition or any other physical or mental illness, had any physical disease condition, took regular medication for any health condition, or had a mental disorder that they had been diagnosed with in the past. Individuals who regularly (i.e., four days or more per week) smoked or drank alcohol were excluded. Additionally, pregnant or lactating women or those having undergone hormone replacement therapy were not recruited. Participants with hearing or vision impairments were not allowed to participate, while those who had fair vision with spectacles were eligible for inclusion in this study. Further details of the participants’ demographics are given below in **Table 1**.

**Table 1:** Demographic details of the study participants.

Participant characteristics	Overall sample (*N* = 74)	BP groups		
		Normotensives (*n* = 33)	Prehypertensives (*n* = 21)	Hypertensives (*n* = 20)
Age range (years)	18-64	18-48	18-54	21-64
Mean Age ± *SD* (years)	28.31 ± 9.12	24.42^a^ ± 6.73	30.10^ab^ ± 9.88	40.25^b^ ± 13.47
Gender	40 males, 34 females	11 males^a^, 22 females^a^	16 males^b^, 5 females^b^	13 males^ab^, 7 females^ab^
Education (years)	18.49 ± 2.16	16.09^a^ ± 1.96	16.43^a^ ± 2.66	16.10^a^ ± 2.45
SBP (mmHg)	113.11 ± 23.23	103.11^a^ ± 10.91	125.51^b^ ± 7.23	143.11^c^ ± 6.00
DBP (mmHg)	75.59 ± 13.93	70.80^a^ ± 5.73	81.80^b^ ± 5.07	90.88^c^ ± 3.60

^a, b, c,^ Common superscripts indicate no significant difference between means.

As evident from **Table 1**, there were significant differences in the mean ages of the three BP groups, *F*(2, 71) = 16.176, *p* <.001, such that hypertensives were significantly older than the normotensives (*p* <.001) as well as the prehypertensives (*p* = .004). Normotensives did not differ significantly from prehypertensives in terms of mean age (*p* = .127). There were also significant differences in the gender composition of the three BP groups, *χ* ^2^(2, N = 74) = 10.813, *p* = .004. Normotensives had significantly lower number of males and significantly higher number of females than the prehypertensives. Additionally, there was no significant difference in the means of education (in years), *F*(2, 71) = 0.158, *p* = .854, across all the three BP groups. By design, SBP and DBP differences were significant across BP groups, *F*(2, 71) = 132.92, *p* <.001; *F*(2, 71) = 101.86, *p* <.001, respectively. The hypertensives had the highest means, for both SBP and DBP, followed by that of the prehypertensives, and that of the normotensives, as expected.

### Tools and measures

#### Omron HEM-7120 fully automatic digital blood pressure machine

Participants’ BP was measured using the Omron HEM-7120, which is a fully automatic digital blood pressure monitor and has been validated in accordance with the European Society of Hypertension International Protocol (ESH-IP) revision 2010 (35). Over the course of two days, four BP readings each were taken, with a two-minute gap between two successive measurements. Since the initial BP reading is usually higher than subsequent ones, especially when measured using an automated BP monitor (36), the first reading on both days was discarded.

#### Facial action coding system

The Facial Action Coding System (FACS) is a comprehensive, anatomically based system developed to objectively classify facial movements by their appearance on the face. Initially, it was developed by Ekman and Friesen (37) and further revisions were made by Ekman, Friesen and Hager (38). FACS identifies visible facial muscle movements called Action Units (AUs), that correspond to specific facial muscle activities. In addition to the 32 atomic facial muscle actions, known as Action Units (AUs), the revision includes 14 extra Action Descriptors (ADs) that take into consideration head posture, gaze direction, and other actions like bite, blow, and thrust of the jaw. using FACS, any facial expression can be objectively described as a combination of AUs.

The intensity of an action is denoted by the letters A, B, C, D, and E. Each letter was later replaced with a corresponding numerical value (1–5) to enable quantitative analyses of intensity (1 being minimal, 3 being moderate, and 5 being extreme). These letters, such as 4B or 4E, are written right after the AU number (here, AU4) to show how much of the overall appearance change that the AU may have caused is actually present. The scale of evidence of an AU’s presence, which establishes the thresholds or criteria for scoring intensity levels, correlates with the five-point FACS intensity notation. In general, the A level denotes a trace of the action; B denotes slight evidence; C denotes marked or pronounced evidence; D denotes severe or extreme evidence; and E denotes maximum evidence (38). For instance, prototypical combination of AUs for happiness are AU6+AU12+AU25. Based on such pre-established prototypical AU combinations for different emotions, the emotion portrayed is labelled.

#### OpenFace: an open-source facial behavior analysis toolkit

OpenFace, an open-source tool, is designed for researchers studying computer vision and comprehending machine learning, the affective computing community, and other individuals interested in creating interactive applications using facial behavior analysis. It was developed by Baltrusaitis, Robinson and Morency (39) at Carnegie Mellon University (CMU), USA. This toolkit is completely free and also resolves all the undue problems that were occurring with the previous toolkits. Many facial behavior analysis tools prior to OpenFace were either very expensive, had unknown algorithms, and often unknown training data; for instance, Affdex (40), FACET (41), and OKAO (42), or did not have complete source code available, which limited their application in real-world or experimental settings. These tools were hard to incorporate into real-time applications, frequently required proprietary hardware, and only offered binary executables. The developers of OpenFace aimed to bridge this gap by offering not just pre-trained models but also full training, understanding and fitting source code. Their approach built upon recent state-of-the-art research including Conditional Local Neural Fields (CLNF) and innovations in action unit detection and eye-gaze estimation.

In addition to being the first open-source tool for facial behavior analysis, OpenFace exhibits cutting-edge capabilities in head pose tracking, eye gaze estimation, AU recognition, and facial landmark detection. Additionally, it can execute all of these functions simultaneously. Among OpenFace’s primary contributions are: 1) the implementation and extension of cutting-edge algorithms; 2) an open source tool that includes model training code; 3) ready-to-use trained models; 4) real-time performance without the need for a Graphical User Interface; 5) a messaging system that makes it simple to implement real-time interactive applications; and 6) availability as a command line tool (for Ubuntu, Mac OS X, and Windows) and a Graphical User Interface (for Windows) (39).

This study utilized OpenFace only as a supportive and validation tool for the facial expression codings (for both accuracy and intensity of expression). Facial expressions were coded by Meenakshi Shukla (one of the first authors), who is formally certified in the FACS through the Paul Ekman Group, the official training and testing body established by Paul Ekman, Wallace V. Friesen, and Joseph C. Hager. Certification requires achieving an accuracy threshold of at least 70–85% on the FACS Final Test, demonstrating proficiency in identifying and coding facial AUs.

The study utilized OpenFace as a supportive and convergent tool, not as a replacement for human coding. The certified FACS coder served as the primary measurement source for both accuracy and intensity, while OpenFace provided independent, AU-level estimates to examine whether an automated system would reproduce similar emotion patterns. Automated outputs were not used to overwrite or adjudicate human ratings; rather, they served to assess convergence and inter-method reliability.

Human FACS coding was prioritized because OpenFace performance can be affected by image tilt, resolution, and ethnic facial morphology, especially in Asian samples (43). Therefore, OpenFace results were interpreted cautiously and used primarily to gauge agreement trends and tool robustness under real-world imaging conditions.

#### Medical comorbidity questionnaire and informed consent form

The medical comorbidity questionnaire was aimed to screen suitable participants for the study by ensuring that they did not meet the exclusion criteria (as listed above in the *Participants* section). The questionnaire asked the demographic details of the participants, such as age, sex, contact details (phone number and email id), formal educational qualification (in years), date and time of taking the questionnaire. After that, past medical history, present medical condition, and family medical history were sought to comprehend their medical record of any illness. Some specific health-related queries were directed towards women participants only, including their pregnancy, lactation, or hormonal therapies. Finally, questions related to smoking and drinking habits were asked to understand whether the participants meet the inclusion criteria. If a participant’s response to the medical comorbidity questionnaire indicated that they were eligible for inclusion in the study, an informed consent form was given to them to read and sign, which included items explaining voluntary participation, confidentiality of the information provided by the individuals, their consent for clicking their photographs, and their right to withdraw from the study and have their data deleted within a particular time frame, without any penalty or consequences.

#### Emotional expressions capturing

For this recording, a cell phone named Infinix Note 12 pro (model no: Infinix X676B) with front camera of 16 MP was used to click selfies and was provided by the researchers to the participant. Participants were given a list of six basic emotions of happiness, sadness, fear, anger, surprise, and disgust (44), as well as neutral. They were instructed to take as many pictures as they wanted for each of the six basic and a neutral emotion, showing their full intensity, until they were satisfied that the clicked image showed the most extreme intensity of that emotion they could express. They were instructed to keep their hand straight at eye level to get a more natural picture.

### Procedure

The study took place over a period of two days for each participant. On the first day, when the participant arrived in the research lab, the researcher made the participant sit on a comfortable chair, which was adjusted to ensure their feet reached the ground and lay flat. Rapport was established with the participant while they relaxed for some time in the chair. This was done to ensure that their BP normalized. Participants were then given an information sheet to read, which explained the study’s purpose, requirements, potential benefits, and any consequences of participating. They were encouraged to ask questions and get any doubts they may have cleared. If they decided to participate, they were given the informed consent form to fill and sign and were thereafter asked to fill out the medical comorbidity screening questionnaire. In order to make sure no questions were left unanswered, the completed forms were promptly reviewed, and participants were politely asked to complete any left-out items, if needed.

Before the participants’ BP was measured, they were told to sit up straight with their feet flat on the floor, their legs uncrossed, and their thighs and lower legs in a 90-degree angle with each other. Throughout the process they were asked to maintain their composure and not speak. Their non-dominant arm was held, with support, at heart level while their BP was measured. Four BP readings were taken on the first day with a 2-min interval between successive readings.

After the four BP readings, the participants were asked to rest for 2–3 minutes. Then, the emotional expression assessment was explained to them. They were informed that a cell phone would be given to them along with a list mentioning the names of six basic emotions (happiness, sadness, anger, fear, disgust and surprise) as well as neutral emotion. The researcher informed them to think about the situations one by one which would give rise to a high/extreme intensity of one of the listed emotions in them. These could include events from their real life, so that the pictures clicked become more realistic. In case, a participant expressed less or no clarity on what a particular emotion was, they were suggested example situations where these might arise in general, such as disgust arising from viewing filth and garbage. They were then asked to take as many as clicks for each emotion as they wanted, and only save the one that they were confident about as being the most intense one. After giving all these instructions, the researcher went outside the lab so that the participant could take the selfies without hesitation. When the clicks were done, the participant called the researcher and handed over the phone to the researcher. Then the participants were asked to come the next day for the remaining four BP measurements. Participants were debriefed then and thanked for their participation. Then, the facial emotion photographs were coded by the certified FACS coder and were also analyzed using OpenFace.

### Statistical analyses

Normality tests were first conducted to ensure the suitability of data for running parametric statistical tests. Kolmogorov-Smirnov and Shapiro-Wilk tests were both conducted to get a clear picture of the dataset, along with skewness and kurtosis values. The admissible range of skewness and kurtosis for conducting analyses of variance is considered to be ±2 (45, 46).

For the facial expression coding process, seven photographs per participant (numbered 1 to 7) were provided by the researcher collecting the data (Shatabdi Bhowmick). Each image was cropped such that the face occupied approximately three-fourths of the photo, ensuring that facial expressions, muscle movements, and wrinkles were clearly visible for analysis. The certified human coder then manually coded all 45 Action Units (AUs) defined by the FACS. Each AU was scored on an intensity scale from 0 (absent) to 5 (maximum intensity), in line with standard FACS guidelines. Based on the pattern of AU activation, the coder also inferred the prototypical emotion expressed in each image.

To supplement the manual coding and compute inter-rater agreement, an automated facial coding software, OpenFace, was used. The images were processed individually through OpenFace, which returned AU intensity scores as floating-point values. These were extracted from the output.csv files and paired with the human-coded data for comparison to assess the consistency and reliability of AU detection.

Because our outcomes included categorical labels (intended-emotion accuracy) with unbalanced prevalence and continuous intensity scores, we reported a panel of complementary inter-rater agreement indices to provide a comprehensive reliability assessment between the human coder and OpenFace. Specifically, 1) Cohen’s Kappa (κ) was used to measure categorical agreement between the two coders, correcting for chance agreement (47); 2) Percent Agreement was calculated to indicate the raw proportion of AUs with matching scores between the coders; 3) Gwet’s AC1 statistic was included to provide a more stable reliability estimate than Kappa, especially in the presence of marginal imbalances (48); 4) Krippendorff’s Alpha (ordinal version) was used to account for ordinal AU intensity scales and potential missing values, offering a robust measure of coder agreement (49); 5) Intraclass Correlation Coefficient (ICC[3,1]) assessed the consistency of AU intensity ratings across coders, focusing on absolute agreement in a fixed-effects model (50, 51). This combination was selected to reduce the risk that the assumptions of any single metric (e.g., κ’s dependence on prevalence or marginal distributions) would disproportionately influence interpretation, thereby ensuring a balanced evaluation of reliability across categorical and continuous outcomes.

To interpret agreement statistics, established benchmarks were used. For ICC(3,1), values below 0.5 indicate poor reliability, values between 0.5–0.75 indicate moderate reliability, 0.75–0.90 indicate good reliability, and values above 0.90 indicate excellent reliability (50). For Cohen’s Kappa and Gwet’s AC1, values above 0.60 were considered acceptable, with values above 0.80 considered strong agreement (48, 52). Krippendorff’s Alpha values above 0.667 are typically regarded as acceptable, while values above 0.80 are considered good (53).

These analyses allowed for a comprehensive assessment of the level of agreement and accuracy in automated versus human-coded facial expression data. These statistics were calculated using Python codes in Anaconda software. Additionally, mean expression intensity scores were computed for both human and OpenFace codings using emotion-specific AU groupings (e.g., AU06, AU12, AU25 for Happiness). Three indices were derived: the mean of all relevant AUs, the mean of only active (non-zero) AUs, and the maximum AU intensity per expression. For simplicity of description and comparison, only the means of active (non-zero) AUs have been mentioned and discussed in the main text. For the mean of all relevant AUs and the maximum AU intensity per expression, see **Supplementary Table 1**.

It is to be carefully noted that all emotion labels were based solely on human coding, as the certified FACS coder was considered the gold standard. OpenFace outputs were used to evaluate the degree of correspondence in AU intensity values, not for emotion classification. Further, only such trials were retained where the intended emotion (i.e., the emotion told to the participants to facially express) matched the emotion label given by the certified FACS coder (who was blind to the intended emotions instructed for each facial expression).

To examine group-level differences in demographic and study variables across the three BP groups, a series of statistical tests were conducted. A chi-square test of independence was performed to assess whether gender distribution differed significantly across the BP categories. For continuous variables such as age, years of education, SBP, and DBP, one-way analyses of variance (ANOVAs) were conducted, followed by Bonferroni-corrected *post hoc* comparisons where appropriate. To explore how SBP and DBP relate to the accuracy of emotional expression as continuous variables, partial bivariate correlations (controlling for age, gender, and education) were carried out. To investigate group differences in the primary outcomes, namely, accuracy of emotional expression and intensity of expressed emotions, separate one-way analyses of covariance (ANCOVAs) were conducted, controlling for age, gender, and education as covariates.

### Why human FACS coding was prioritized over automated OpenFace coding?

While OpenFace offers a powerful and automated approach to facial AU detection, several limitations necessitate a more cautious interpretation of its outputs. Research suggests that OpenFace coding, being algorithmically generated from pixel-level facial features, can sometimes detect subtle AUs that may not be easily perceptible to the human eye (39). However, such detections may occasionally lack contextual or interpretive nuance, particularly in emotionally ambiguous or culturally nuanced expressions. For instance, OpenFace’s performance has been shown to degrade significantly when analyzing faces of Asian individuals and children, likely due to imbalanced training datasets across racial and age groups (43). Moreover, OpenFace’s efficiency is highly sensitive to image quality: its accuracy declines as the resolution of input images worsens—a critical concern in real-world settings such as surveillance or low-light environments (43).

In addition, although OpenFace is appreciated for its compact size and ease of integration on resource-limited systems, it generally exhibits lower accuracy than other facial recognition models and requires extensive preprocessing (54). Such preprocessing demands, including face alignment and normalization, can also limit real-time applicability and introduce additional sources of variability. Given these limitations, and considering that the human coder in the present study was a Certified FACS Expert trained to interpret subtle, complex, and context-dependent AU combinations, greater weight was assigned to the manual coding. Human coders not only integrate perceptual sensitivity but also apply theoretical and contextual knowledge to produce more reliable emotion judgments, particularly in nuanced or culturally variable expressions.

## Results

**Figure 1:**
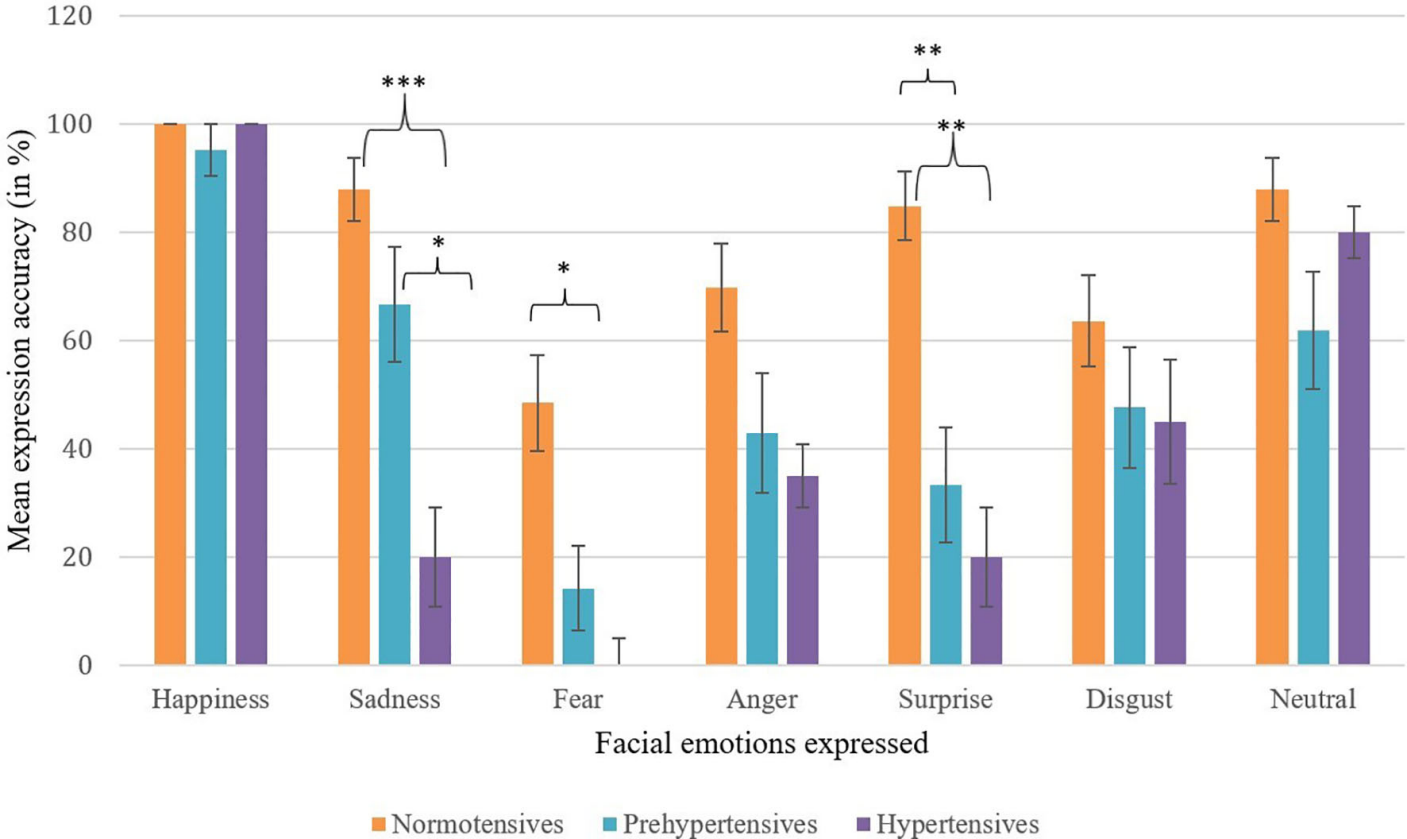
Mean expression accuracy across facial emotions by blood pressure group. Error bars represent ±1 SE. Selective dampening is evident for sadness, fear, and surprise among prehypertensive and hypertensive groups. Asterisks indicate statistically significant between-group differences (*p <.05, **p <.01, ***p <.001).

Normality tests revealed normal or near-normal distribution of data, justifying the use of parametric statistics for analyses. Skewness values for the data ranged from -.767 to 1.888 while kurtosis values ranged from -1.523 to 1.884, both of which lied within the recommended range of ±2 (45, 46). To begin with, treating BP as a categorical variable with three distinct groups of normotensives, prehypertensives, and hypertensives, the accuracy of emotional expression was compared across the three BP groups for each of the six basic and a neutral emotion using one-way ANCOVAs (controlling for covariates age, gender, and education). A significant effect of BP groups on accuracy of emotional expressions was obtained for the emotion categories of sadness, but not for anger or disgust. Rather the effect of BP groups was found significant for the accuracy of expressing fear and surprise (see **Table 2**, **Figure 1** for means). Given that the groups did not differ significantly on neutral expression and neither SBP nor DBP correlated significantly with the accuracy of neutral expressions (baseline), the findings taken together suggest that the emotion of happiness is robust and resistant to the deteriorating effects of BP on emotional expression while other emotional expressions seem to be affected by increase in BP.

**Table 2:** Mean accuracy of emotional expression (in percentages) across different BP groups and emotion categories, along with F and effect size.

Emotion categories	BP groups			*F*(2, 68)	Partial eta squared
	Normotensives	Prehypertensives	Hypertensives		
Happiness	100.00	95.24	100.00	.913	.026
Sadness	87.88^a^	66.67^a^	20.00^b^	7.571***	.182
Fear	48.48^a^	14.28^ab^	0.00^b^	4.692*	.121
Anger	69.70	42.86	35.00	1.116	.032
Surprise	84.85^a^	33.33^b^	20.00^b^	8.620***	.202
Disgust	63.64	47.62	45.00	.053	.002
Neutral	87.88	61.90	80.00	2.607	.071

*p < .05, ***p < .001

Common superscripts indicate no significant difference between means and are shown only for means where overall F was significant.

**Figure 2:**
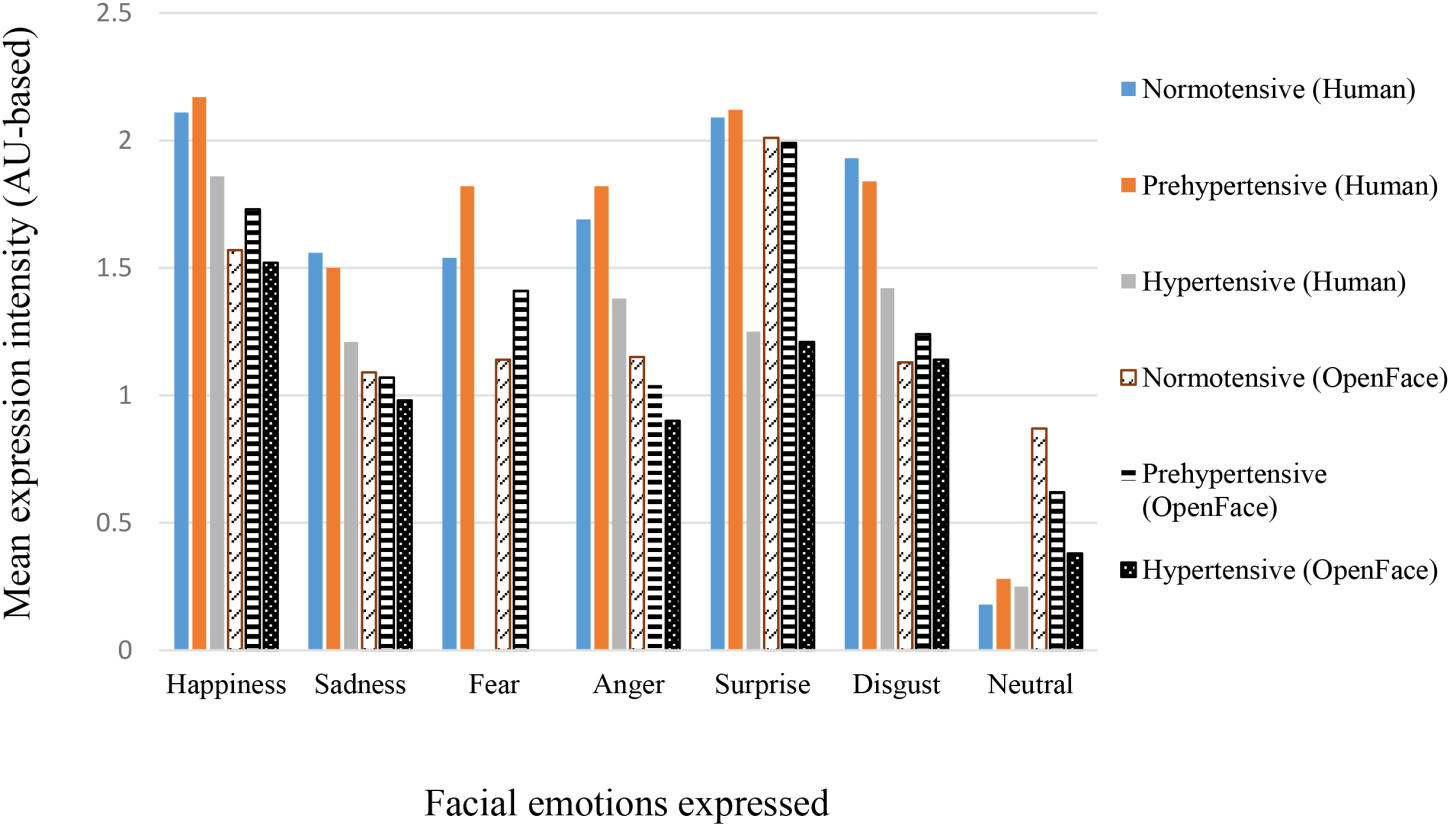
Mean expression intensity across blood pressure groups for Human and OpenFace codings. Values represent group means (see **Table 4** for SDs).

**Table 3** summarizes the mean percent agreement, inter-rater reliability statistics, and expression intensity scores (Human and OpenFace) for correctly expressed trials across the six basic emotions and neutral, separately for the three BP groups (see also **Figure 2** for expression intensity scores). Across emotions, mean percent agreement between human and OpenFace codings was generally high (70.23–89.42%), with the highest values observed for Surprise and Neutral in the normotensive group, and the lowest for Disgust in the hypertensive group. However, agreement statistics varied notably across emotion categories and groups.

**Table 3:** Mean percent agreement, inter-rater reliability statistics, and expression intensity scores (human and OpenFace) for the six basic and neutral emotions across BP groups.

Emotion	BP groups	Mean % sgreement ± SD	Mean Cohen’s kappa ± SD	Mean Gwet’s AC1 ± SD	Mean Krippendorff’s alpha ± SD	Mean ICC(3,1) ± SD	Mean human intensity (active AUs) ± SD	F	Mean OpenFace intensity (active AUs) ± SD	F
Happiness	Normotensive (N = 33)	85.19 ± 7.12	.50 ± .19	.71 ± .21	.62 ± .33	.64 ± .18	2.11 ± .54		1.57 ± .59	
	Prehypertensive (N = 20)	80.46 ± 17.29	.41 ± .25	.47 ± .34	.47 ± .33	.52 ± .35	2.17 ± .79	.653	1.73 ± .54	.789
	Hypertensive (N = 20)	80.98 ± 11.03	.42 ± .22	.57 ± .27	.43 ± .32	.50 ± .34	1.86 ± .83		1.52 ± .74	
Sadness	Normotensive (N = 29)	86.07 ± 7.00	.47 ± .23	.70 ± .22	.53 ± .31	.52 ± .23	1.56 ± .53		1.09 ± .45	
	Prehypertensive (N = 14)	83.95 ± 6.76	.41 ± .19	.56 ± .26	.49 ± .20	.50 ± .18	1.50 ± .66	1.171	1.07 ± .36	.515
	Hypertensive (N = 4)	76.66 ± 16.89	.28 ± .18	.26 ± .22	.04 ± .50	.46 ± .25	1.21 ± .16		.98 ± .28	
Fear	Normotensive (N = 16)	88.61 ± 3.80	.55 ± .20	.75 ± .17	.72 ± .13	.68 ± .16	1.54 ± .30		1.14 ± .41	
	Prehypertensive (N = 3)	88.90 ± 6.67	.58 ± .20	.72 ± .30	.63 ± .20	.62 ± .14	1.82 ± .69	.151	1.41 ± .43	.730
	Hypertensive (N = 19)	---	.75 ± .17	---	---	---	---		---	
Anger	Normotensive (N = 23)	87.92 ± 5.18	.47 ± .28	.73 ± .24	.57 ± .40	.46 ± .31	1.69 ± .67		1.15 ± .63	
	Prehypertensive (N = 9)	83.95 ± 6.06	.39 ± .27	.59 ± .34	.48 ± .35	.44 ± .32	1.82 ± .80	.957	1.04 ± .32	2.017
	Hypertensive (N = 7)	75.50 ± 17.87	.37 ± .25	.54 ± .27	.39 ± .28	.44 ± .24	1.38 ± .55		.90 ± .28	
Surprise	Normotensive (N = 28)	88.92 ± 4.74	.55 ± .21	.76 ± .20	.67 ± .21	.68 ± .19	2.09 ± .62		2.01 ± .76	
	Prehypertensive (N = 7)	81.26 ± 8.50	.43 ± .16	.52 ± .31	.42 ± .43	.70 ± .20	2.12 ± .69	2.168	1.99 ± .84	2.694
	Hypertensive (N = 4)	82.24 ± 20.46	.29 ± .22	.41 ± .32	.39 ± .29	.39 ± .29	1.25 ± .96		1.21 ± .83	
Disgust	Normotensives (N = 21)	84.42 ± 7.16	.45 ± .28	.75 ± .11	.58 ± .29	.46 ± .27	1.93 ± .56		1.13 ± .55	
	Prehypertensives (N = 10)	74.00 ± 8.45	.30 ± .21	.37 ± .29	.28 ± .46	.34 ± .28	1.84 ± .82	1.876	1.24 ± .76	.106
	Hypertensives (N = 9)	70.23 ± 30.22	.35 ± .38	.35 ± .54	.44 ± .36	.43 ± .32	1.42 ± .74		1.14 ± .70	
Neutral	Normotensives (N = 29)	89.42 ± 8.88	.27 ± .38	.58 ± .42	.42 ± .55	.33 ± .49	.18 ± .41		.87 ± .52	
	Prehypertensives (N = 13)	88.38 ± 7.87	.31 ± .34	.47 ± .38	.45 ± .54	.40 ± .48	.28 ± .45	.303	.62 ± .51	2.592
	Hypertensives (N = 16)	88.33 ± 10.81	.34 ± .41	.49 ± .44	.46 ± .51	.32 ± .76	.25 ± .55		.38 ± .39	

Only trials where the intended emotion matched the human-coded emotion were included in this analysis. Dashes indicate that no emotional expression was accurate for the emotion of fear in the hypertensive group.

Cohen’s Kappa values ranged from .29 to .75, indicating predominantly poor to moderate agreement according to conventional benchmarks. Gwet’s AC1 values were generally higher than Kappa for the same conditions. Krippendorff’s Alpha values showed a similar pattern to Kappa, with generally lower values for the hypertensive group across several emotions, suggesting greater variability in coding agreement in this group. ICC(3,1) values also indicated modest reliability, typically between .32 and .70, with the highest ICCs for Surprise (prehypertensives) and the lowest for Neutral in hypertensives.

Mean human-coded expression intensity scores were consistently higher than the corresponding OpenFace intensities across emotions and BP groups. Happiness and Surprise generally elicited the highest intensity scores, whereas Neutral showed the lowest. In most cases, hypertensive participants produced lower intensity expressions than the normotensive and prehypertensive groups, for both human and OpenFace coding. However, one-way ANCOVAs revealed no significant difference across BP groups in either human or OpenFace coded intensities. This is more likely because of the wide variations in the number of individuals (see *N* in **Table 4**) per BP group who correctly expressed the facial emotions. Taken together, these results indicate that while raw percent agreement between human and automated coding was high, chance-corrected metrics such as κ revealed more modest concordance, particularly for certain emotions and in hypertensive participants.

**Table 4:** Partial bivariate correlations (controlling for age, gender, and education) of SBP and DBP with accuracy of emotional expressions across the six basic and neutral emotions.

BP	Emotion categories						
	Happiness	Sadness	Fear	Anger	Surprise	Disgust	Neutral
SBP	.076	-.426***	-.123	-.430***	.033	-.456***	.003
DBP	-.054	-.348**	-.003	-.344**	-.041	-.310**	-.129

**p < .01; ***p < .001.

Next, partial correlations of SBP and DBP with the accuracy of emotional expressions revealed significant negative correlations of both SBP with DBP with the accuracy of expressing sadness, anger, and disgust (see **Table 4**), indicating that with increase in either SBP or DBP, the facial expressions of sadness, anger, and disgust, prominent negative emotions (with the exception of fear), deteriorates.

Thus, the results of correlational analyses were similar to group comparison analyses with respect to sadness, though these contrasted with group comparisons for the emotions of anger and disgust.

## Discussion

This study is the first to test whether cardiovascular emotional dampening (CED) also affects the production of facial expressions, using both certified FACS coding and automated AU detection (OpenFace). Across analyses, the findings converged on three principal patterns. First, elevated BP was linked to reduced accuracy in producing certain negative emotions, most consistently sadness, with additional decrements for fear and surprise in group-level comparisons. Positive affect expressions such as happiness were largely preserved, suggesting that CED in expression may be selective rather than uniform across emotions. Second, expression intensity followed the same directional trend as accuracy, with prehypertensive and hypertensive participants showing generally weaker displays, particularly for sadness. These differences did not reach statistical significance, which likely reflects limited statistical power after restricting analyses to correctly produced expressions. Third, OpenFace showed moderate to strong agreement with the certified FACS coder in the normotensive group, but this correspondence weakened with higher BP, consistent with reduced or ambiguous expressivity. Together, these results indicate that elevated BP is linked to both lower accuracy and reduced clarity of emotional output. Thus, CED in elevated BP is reflected not only in the perception of others’ emotions, as documented previously (1, 2, 4–6, 10, 11, 55), but also in the ability to produce one’s own facial expressions with reliability and vigor. Moreover, the pattern of selective impairment, along with reduced correspondence between automated and human coders in groups with higher BP, reinforces the importance of expert human FACS coding when investigating nuanced changes in emotional expressions.

The present findings extend the CED framework from perception to production of facial emotions. Prior work has largely shown that individuals with higher resting BP exhibit reduced sensitivity when recognizing or evaluating others’ emotions across visual and auditory modalities (e.g., 1, 3–6). Here we show that a similar pattern emerges when people are asked to generate facial expressions themselves. Two complementary patterns emerged. First, group-level ANCOVAs indicated that the accuracy of posed expressions declined with increasing BP status, most consistently for sadness, and, though less robustly, for fear and surprise. Second, correlational analyses controlling for age, gender, and education revealed a somewhat broader pattern, with higher systolic and diastolic BP associated with lower accuracy in expressing sadness, anger, and disgust. Thus, sadness emerged as the most consistent deficit across both analytic approaches, while other negative emotions showed BP-related impairments that were either group-specific (fear, surprise) or association-specific (anger, disgust).

A notable feature is that BP effect was selective: happiness was largely preserved, while sadness, and to a lesser extent fear and surprise, showed the strongest dampening. In correlational findings, sadness, anger, and disgust were seen dampened. This selective pattern is compatible with two ideas already present in the CED literature. First, the “generalized dampening” view (e.g., 3) does not preclude unequal effects across discrete emotions; it simply predicts that the direction of change is toward reduced emotional responsiveness. Second, although the current study focuses on production rather than recognition, similar patterns have been observed in perception-based tasks. For instance, Jain, Shukla, and Pandey (8) reported that, even within the normotensive range, higher SBP and DBP were negatively correlated with facial emotion recognition accuracy across multiple emotion categories. While their linear correlations were strongest for happiness, moderate negative associations were also noted for negative non-aroused emotions (sadness, disgust) and, to a lesser extent, for negative aroused emotions. Partial-least-squares models showed that elevated BP predicted dampening for both positive and non-aroused negative emotions. The overlap in affected emotion categories, particularly negative non-aroused emotions, suggests that the same BP-linked mechanisms that impair emotion recognition may also extend to the generation of emotional expressions, offering indirect support for the observed correlations in the present study. Our finding that happiness is least affected also aligns with reports of preserved processing for approach-oriented positive affect in some elevated-BP samples (11).

The present findings extend recognition-based evidence of CED by showing that facial production is also attenuated. Production and recognition draw on overlapping perception–action systems (mirror-neuron/simulation accounts). Observing and experiencing disgust recruit shared insular circuits (27). Individuals who can’t produce expressions show poorer recognition (Moebius syndrome: 28), and disorders with impaired facial output likewise show recognition deficits (schizophrenia; Parkinson’s: 29–31). In healthy observers, blocking mimicry impairs recognition, whereas training mimicry improves it (32, 33). Taken together, this literature justifies the inference that our reduced expression accuracy likely co-occurs with poorer recognition (as reported in CED studies) via at least partly shared mechanisms.

Apart from the above core findings, we observed that the inter-rater agreement between the certified FACS coder and OpenFace was higher in normotensives and dropped in prehypertensive and hypertensive groups. We see two complementary explanations. First, when participants produce weaker, more ambiguous AU patterns (lower intensity, partial activation, or brief/unstable configurations), automated detectors, which rely on pixel-level features and learned thresholds, are more likely to underrate or miss subthreshold activations. Human FACS coders, by contrast, can integrate contextual cues (e.g., the co-occurrence of AUs, asymmetries, or wrinkle patterns) to infer an intended expression. This helps explain why OpenFace underestimated intensity relative to the human coder across groups. Second, agreement coefficients (κ, α, ICC) are sensitive to prevalence and range. Sparse positives (many zeros) and restricted intensity ranges depress κ (the “kappa paradox”) and can pull ICC downward even when raw percent agreement seems respectable (56, 57). Because elevated BP groups generated fewer high-intensity activations for negative emotions, the statistical ceiling for agreement was lower, amplifying apparent human–machine discrepancies. These patterns argue, practically, for giving greater weight to certified human coding when the research question hinges on subtle, low-intensity, or clinically variable expressions. Automated tools remain valuable, especially for scale, reproducibility, and AU-level transparency. However, their limitations should be acknowledged when signal strength is low or when datasets differ from training corpora in age, race/ethnicity, camera geometry, or image quality.

Some of the inter-rater statistics fell in the modest range, particularly in prehypertensive and hypertensive groups. Two clarifications are important for interpretation. First, modest agreement coefficients are expected when expressions are weak and infrequent, since even small discrepancies inflate κ and α. Second, our analytical choice to report intensity means only for trials in which the human coder identified the target emotion is statistically conservative. This approach protects construct validity for “intensity of the intended expression,” but it reduces N, especially in higher-BP groups, lowering stability and widening confidence intervals. The directional consistency of effects across indices (lower accuracy and lower intensity with higher BP) suggests a coherent phenomenon despite these constraints.

The selective dampening we observed in facial expression production is compatible with proposals that baroreflex-related central pathways modulate affective responsiveness by influencing cortical and subcortical circuits involved in emotional motor output (25). Sustained baroreceptor activation can dampen cortical responses to affective and pain stimuli, lowering the neural “gain” that drives facial expression. While prior baroreflex research has not differentiated by emotion category, our data suggest that such modulation may be especially evident for low-arousal negative emotions such as sadness. In contrast, some behavioral findings in hypertensive samples point to relative preservation or enhancement of threat-related processing, including an anger-recognition bias (11, 58), raising the possibility of state-dependent gating in which threat-detection and mobilization circuits are spared or sensitized. This two-process view, i.e., general dampening alongside selective preservation, offers one potential explanation for our pattern: sadness (and sometimes fear) is most reduced, happiness is relatively preserved, and anger is not uniformly blunted.

Although the present study did not directly assess physiological or motor processes, the pattern of reduced expressive accuracy and intensity among individuals with elevated BP can be interpreted within embodiment and facial-feedback frameworks. These theories posit that emotional experience and expression are maintained through reciprocal interactions among facial musculature, autonomic arousal, and affective appraisal (16, 20, 21, 24). Prior work indicates that elevated blood pressure is associated with reduced autonomic flexibility and blunted physiological reactivity to affective stimuli (25, 26). Viewed together, these perspectives suggest that the expressive dampening observed here may involve attenuated physiological feedback mechanisms that normally amplify and differentiate affective motor output. These may reflect partial involvement of the same physiological mechanisms that underlie CED in recognition tasks. Such physiological dampening could, in turn, weaken the sensory–motor feedback loops that help sustain vivid and differentiated emotional expressions. While this interpretation remains inferential, it offers a coherent theoretical link between cardiovascular regulation and expressive aspects of emotional functioning, extending existing models of cardiovascular emotional dampening beyond perception to production.

Although earlier work has suggested that the relationship between blood pressure and emotional dampening may be non-linear (8), with moderate elevations associated with optimal emotion recognition and both higher and lower levels of BP linked to reduced affective responsiveness, our findings did not reveal clear evidence for such a curvilinear trend. Given the relatively narrow BP range in the present sample, this possibility remains open and should be evaluated in future studies using larger, population-based samples that encompass a broader BP continuum. Such investigations would help clarify whether emotional expressivity follows an inverted-U or threshold trajectory across the hypotensive–hypertensive spectrum.

The present study has several notable strengths. First, we employed a dual-method coding approach, combining FACS ratings by a certified human coder with automated OpenFace analysis at the level of individual AUs. Second, expression intensity was summarized using *a priori* emotion prototypes, allowing for targeted interpretation of AU patterns in relation to discrete emotions. Third, we assessed inter-method agreement using multiple complementary indices, i.e., percent agreement, Cohen’s κ, Gwet’s AC, Krippendorff’s α, and the intraclass correlation coefficient (ICC), to provide a more robust evaluation of reliability. Finally, our analytic strategy considered both categorical group comparisons (normotensives, prehypertensives, hypertensives) and continuous associations with SBP and DBP, enabling a comprehensive assessment of CED across different levels of analysis.

Despite these strengths, several limitations should be acknowledged. First, the age range of the sample was broad, and the group sizes, particularly for the prehypertensive and hypertensive groups, were modest. Although age and gender were statistically controlled in all analyses, residual confounding due to demographic imbalance cannot be fully ruled out. Prior literature has documented age- and gender-related differences in emotional recognition (e.g., meta-analysis, 59, 60) and expression (61, 62), which may have influenced the magnitude of effects observed. Recruiting clinically verified hypertensive participants willing to engage in laboratory-based facial expression tasks is inherently challenging, which partly explains the uneven group sizes. Future research should therefore aim to include larger, demographically balanced samples to strengthen the generalizability and robustness of these findings. Second, the decision to compute intensity means only for accurately produced expressions improved interpretability but reduced power, particularly for negative emotions in elevated BP groups. Third, posed photographs, while controlled, may under-sample dynamic cues that help both humans and algorithms recognize and produce expressions. Fourth, the study design was cross-sectional, and thus, causal claims about BP and expressivity require longitudinal or experimental manipulation (e.g., pharmacological or baroreflex interventions). Fifth, the study also relied on a single certified FACS coder to ensure consistency, which introduces the possibility of coder bias. Including multiple trained raters in future work would allow estimation of inter-rater reliability and provide a more robust framework for validating automated outputs. Sixth, while this study focused specifically on expressive dampening, future extensions could include both emotion recognition and production measures within the same participants. Such a design would enable a direct test of the hypothesized recognition–expression correspondence in the CED framework. Yet another and crucial limitation could be that participants were asked to imagine the situations that would arouse the emotions in them, and then portray those emotions. Individual differences in imagery capacity could have influenced the accuracy and intensity of the emotions expressed. Moreover, because these expressions were generated in response to verbal instructions rather than real emotional stimuli, they likely relied more on voluntary facial control than on spontaneous affective activation, which may limit ecological validity.

Another limitation concerns the voluntary, posed nature of the expressions. Participants were instructed to imagine emotional situations and deliberately display the corresponding facial expressions, which may not fully reflect the spontaneity or neural underpinnings of naturally occurring emotions. Posed and spontaneous expressions differ in both morphology and generation pathways: spontaneous expressions tend to be less stereotyped, unfold with greater temporal variability, and are more strongly linked to limbic–autonomic activation (63–65). By contrast, posed expressions rely more on voluntary motor control and may engage distinct hemispheric networks (65). This distinction implies that the present findings reflect the ability to reproduce recognizable expressions rather than spontaneous affective responses. Nevertheless, this approach ensured methodological continuity with previous CED studies, where recognition tasks have almost exclusively relied on posed facial expressions rather than naturally occurring affective displays. Employing a parallel paradigm allowed us to examine expressive dampening under comparable, controlled conditions. Recording spontaneous expressions elicited by genuine emotional stimuli, while more demanding, would provide greater ecological validity and help determine whether similar BP-related dampening effects generalize to real emotional contexts.

Although the number of usable observations for certain emotions (e.g., sadness, fear) was smaller in the elevated-BP groups, this reflects the analytic restriction to accurately produced expressions rather than missing participants. Notably, the lower number of participants able to generate these expressions correctly may itself index Cardiovascular Emotional Dampening, consistent with prior evidence that CED manifests more robustly in diminished *accuracy* of emotional recognition than in intensity (e.g., 4, 55). Thus, variability in cell sizes likely mirrors the very dampening effect under investigation, although replication with larger samples will help confirm this interpretation.

Demonstrating that elevated BP is associated with impoverished facial expression has practical and theoretical implications. Social communication relies on both perceiving and sending clear affective signals; dampened production may contribute to interpersonal misunderstandings, blunted social reward, or compensatory reliance on verbal cues. Theoretically, production deficits support involvement of mirror neurons, or at least common physiological mechanisms involved in emotional dampening manifested in recognition and expression of emotions. Future research can build on these findings in several ways. First, employing dynamic expression tasks would allow the capture of onset and offset kinematics, providing a richer temporal profile of emotional displays. Second, incorporating multiple human coders would enable estimation of human–human reliability in parallel with human–machine agreement, offering a more complete reliability framework. Third, experimental manipulations, such as facial-mimicry training or interventions targeting heart rate and blood pressure, could help to test the causal role and reversibility of CED.

While our findings must be interpreted cautiously, they raise intriguing possibilities for psychiatric relevance. In mood disorders, especially depression, emotional expression is often disrupted: patients may experience ambivalence over whether or how to express emotions (66) or show context-insensitive, flattened expressive responses (67). Such expressive disturbances parallel the “dampened” facial output observed here in individuals with elevated BP. Improvements in emotional blunting following a switch from SSRI/SNRI treatment to vortioxetine were shown to predict functional recovery and enhanced motivation in depression patients, underscoring that modulation of affective expression is clinically meaningful (68). In the cardiovascular domain, hypertension has been associated with cognitive impairments (review, 69), diminished autonomic flexibility (70), and mood dysregulation (71, 72), factors which plausibly interact with affective processing deficits. Thus, expressive dampening in elevated BP may overlap mechanistically with affective flattening observed in mood disorders. Although speculative, this intersection invites future research into whether expressive dampening could represent a subclinical affective phenotype in hypertensive individuals, potentially serving as an early marker of vulnerability to depression or cognitive decline.

However, we emphasize that the current results are exploratory; larger, longitudinal, and clinically enriched samples are needed to test whether expressive dampening indeed predicts psychiatric outcomes such as depressive symptoms, emotional blunting, or cognitive impairment.

## Conclusion

In sum, the data indicate that cardiovascular emotional dampening is not confined to the recognition of others’ emotions. Individuals with elevated blood pressure also show reduced accuracy and lower intensity when producing facial expressions, with sadness most affected. Human–automated agreement is highest where expressions are clearest (normotensives) and declines as expressions weaken (prehypertensives, hypertensives), underscoring the continuing value of expert human coding in research on subtle affective signals. These results sharpen the CED construct, linking cardiovascular state to both sides of the social–emotional exchange, recognizing and expressing, and suggest concrete methodological steps for future studies.

## Data Availability

The raw data supporting the conclusions of this article will be made available by the authors, without undue reservation.
