# Supplementary material for: From recognition to expression: extending cardiovascular emotional dampening to facial expressions under elevated blood pressure
Source: Front Psychiatry. 2025 Nov 5;16:1681377. doi: 10.3389/fpsyt.2025.1681377 (PMC12627053; doi:10.3389/fpsyt.2025.1681377)
Supplement: Supplementary file 1 [file Table1.docx]

**Supplementary Table 1**

*Mean intensity of emotions considering all AUs and maximum AU intensity for accurately expressed emotions across blood pressure groups*

| **Emotion** | **BP Group** | **Mean Human Intensity (All AUs) ± SD** | **Mean OpenFace Intensity (All AUs) ± SD** | **Max Human AU Intensity ± SD** | **Max OpenFace AU Intensity ± SD** |
| --- | --- | --- | --- | --- | --- |
| **Happiness** | **Normotensive** | 1.18 ± .89 | 1.17 ± .84 | 2.73 ± .72 | 2.84 ± .66 |
|  | **Prehypertensive** | 1.40 ± .82 | 1.39 ± .85 | 2.95 ± 1.24 | 2.94 ± .64 |
|  | **Hypertensive** | 1.34 ± .77 | 1.61 ± .92 | 2.20 ± .95 | 2.54 ± 1.20 |
| **Sadness** | **Normotensive** | .96 ± .59 | .73 ± .46 | 2.02 ± .63 | 1.98 ± .77 |
|  | **Prehypertensive** | 1.06 ± .64 | 1.07 ± .72 | 1.81 ± .66 | 1.82 ± .50 |
|  | **Hypertensive** | .95 ± .38 | .83 ± .19 | 1.75 ± .50 | 1.98 ± .52 |
| **Fear** | **Normotensive** | .89 ± .64 | .63 ± .42 | 2.19 ± .40 | 2.26 ± .67 |
|  | **Prehypertensive** | 1.22 ± .86 | .77 ± .43 | 2.33 ± .58 | 2.50 ± .61 |
|  | **Hypertensive** | --- | --- | --- | --- |
| **Anger** | **Normotensive** | .86 ± .62 | .56 ± .34 | 2.33 ± .67 | 2.16 ± 1.10 |
|  | **Prehypertensive** | 1.22 ± .68 | .79 ± .55 | 2.02 ± .68 | 1.95 ± .31 |
|  | **Hypertensive** | 1.27 ± .54 | .86 ± .54 | 1.91 ± .24 | 1.67 ± .58 |
| **Surprise** | **Normotensive** | 1.41 ± .90 | 1.50 ± .95 | 2.46 ± .74 | 3.26 ± .85 |
|  | **Prehypertensive** | 2.18 ± .66 | 2.22 ± .85 | 2.43 ± .53 | 3.48 ± 1.09 |
|  | **Hypertensive** | .88 ± .75 | .91 ± .63 | 1.50 ± 1.29 | 2.05 ± 1.58 |
| **Disgust** | **Normotensive** | .97 ± .77 | .58 ± .46 | 2.48 ± .68 | 1.77 ± .92 |
|  | **Prehypertensive** | 1.42 ± .73 | .91 ± .90 | 2.30 ± .63 | 2.04 ± 1.08 |
|  | **Hypertensive** | 1.15 ± .85 | 1.17 ± .89 | 1.74 ± .96 | 1.95 ± 1.04 |
| **Neutral** | **Normotensive** | .10 ± .26 | .38 ± .51 | .21 ± .41 | 1.83 ± .92 |
|  | **Prehypertensive** | .04 ± .08 | .34 ± .44 | .31 ± .48 | 1.33 ± 1.01 |
|  | **Hypertensive** | .19 ± .45 | .23 ± .51 | .46 ± .84 | .80 ± .89 |
